# Magnetization reversal and interlayer exchange coupling in ferromagnetic metal/semiconductor Fe/GaMnAs hybrid bilayers

**DOI:** 10.1038/s41598-018-28882-0

**Published:** 2018-07-12

**Authors:** Kritsanu Tivakornsasithorn, Taehee Yoo, Hakjoon Lee, Sangyeop Lee, Seonghoon Choi, Seul-Ki Bac, Kyung Jae Lee, Sanghoon Lee, Xinyu Liu, M. Dobrowolska, Jacek K. Furdyna

**Affiliations:** 1 Department of Physics, Korea University, Seoul, 136-701 Korea; 2 Department of Physics, Faculty of Science, Mahidol University, Bangkok, 10400 Thailand; 3 Department of Physics, University of Notre Dame, Notre Dame, Indiana 46556 USA

## Abstract

We report a detailed study of magnetization reversal in Fe/GaMnAs bilayers carried out by magnetotransport measurements. Specifically, we have used planar Hall resistance (PHR), which is highly sensitive to the direction of magnetization, and is therefore ideally suited for tracking magnetization as it reorients between successive easy axes in the two magnetic layers during reversal. These reorientations take place separately in the two magnetic layers, resulting in a series of different magnetization alignments (parallel or orthogonal) during reversal, providing a series of stable PHR states. Our results indicate that the magnetic anisotropy of the structure is dominated by cubic symmetry of both layers, showing two in-plane easy axes, but with significantly different energy barriers between the easy orientations. Importantly, a careful analysis of the PHR results has also revealed the presence of strong ferromagnetic interlayer exchange coupling (IEC) between the two magnetic layers, indicating that although magnetization reorients separately in each layer, this process is not independent, since the behavior of one layer is influenced by its adjacent magnetic neighbor. The ability to design and realize multiple PHR states, as observed in this investigation, shows promise for engineering Fe/GaMnAs bilayer structures for multinary magnetic memory devices and related multinary logic elements.

## Introduction

Magnetic multilayers are of special interest in the area of spintronic applications owing to important spin phenomena which they display, such as giant magnetoresistance (GMR)^1,2^ and tunneling magnetoresistance (TMR)^3–5^, that are already being extensively used in spintronic devices. GMR and TMR originate from resistance differences between parallel and antiparallel alignments of magnetization in multilayers, so that manipulation of magnetization alignment in such systems is crucial for the operation of these devices^6–8^. In this context, various types of magnetic multilayers have been designed for controlling magnetic alignment. Most of such multilayers consist of metallic ferromagnetic layers, in which magnetization is controlled either by an external magnetic field, or by current-induced spin transfer torque (STT)^4,5^. However, it is now well established that magnetic properties of the ferromagnetic semiconductor GaMnAs can be controlled by numerous external means, such as gate voltage, magnetic field, strain, and illumination^9–13^. These diverse control methods provide significant advantages for designing multilayer structures, making multilayers based on GaMnAs especially promising for novel device applications.

One promising multilayer structures is the hybrid system consisting of ferromagnetic metal Fe and ferromagnetic semiconductor GaMnAs layers. Recently such Fe/GaMnAs bilayers were investigated^14–18^ with special focus on role of coupling between the Fe and GaMnAs layers, and have already revealed the interesting fact that the Curie temperature *T*_*c*_ of the GaMnAs layer is enhanced in the interface region to room temperature owing to its strong coupling with ferromagnetic Fe^14^. So far investigations of such Fe/GaMnAs bilayers were restricted to measurements using the superconducting quantum interference device (SQUID) and magnetic circular dichroism (MCD)^14–18^. Direct investigation of transport properties of Fe/GaMnAs bilayers is, however, particularly important, since any spintronic devices based on this system will eventually involve electrical transport. Additionally, transport phenomena such as the planar Hall resistance are especially sensitive to the orientation of magnetization in ferromagnetic layers, making them ideal for studies of the effect of magnetization alignment that underlies the operation of devices based on magnetic bilayers.

We note here that analyzing transport measurements carried out on the Fe/GaMnAs bilayers encounter a difficulty that arises from the large conductivity of the metallic Fe layer, which shunts the current through Fe/GaMnAs bilayer, so that the magneto-transport properties of GaMnAs are normally not probed by such measurements. In this study, we are therefore using Fe/GaMnAs structures in which the GaMnAs layer is one order of magnitude thicker than the Fe layer, so as to allow detection of the contribution of the GaMnAs layer to the electrical transport signal observed on this hybrid bilayer.

## Experiments

The Fe/GaMnAs ferromagnetic hybrid structure designed for this investigation was grown by molecular beam epitaxy (MBE) on a (001) GaAs substrate. A 100-nm GaAs buffer layer was first deposited on the substrate at 600 °C, followed by deposition of a 100-nm Ga_1−*x*_Mn_*x*_As film with *x* = 0.06 at 250 °C. The system was then cooled to room temperature for deposition of an 8-nm layer of crystalline Fe. Finally, the structure was capped by a 2 nm Au film to protect the Fe layer from oxidation.

**Figure 1:**
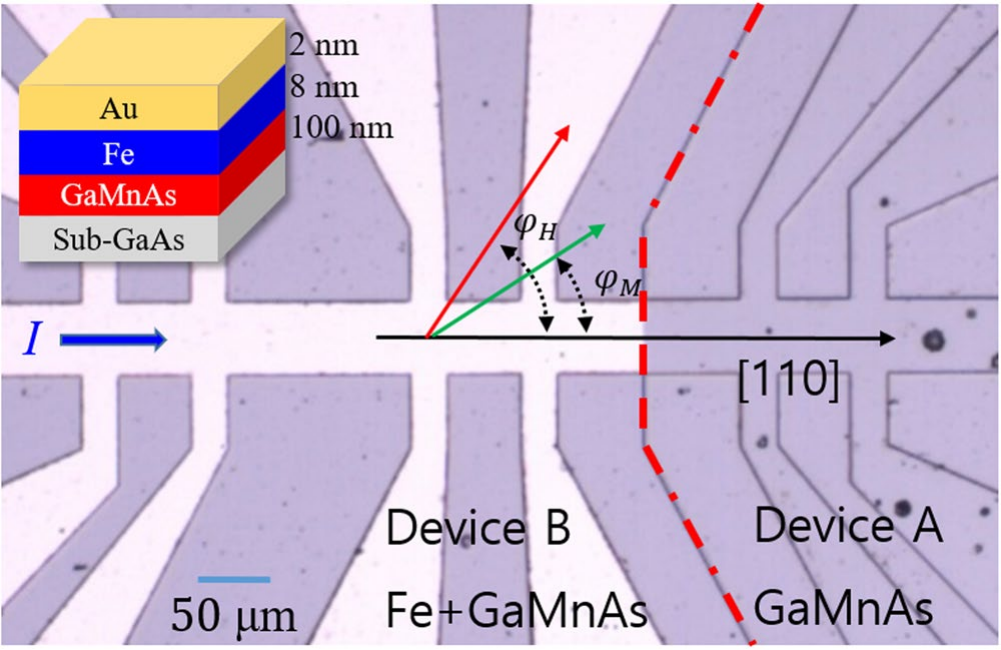
Optical top-view image of Hall device used for PHR measurements. Fe appears as white areas, and GaMnAs (or GaAs) as gray. The red dotted line separates Device B patterned on Fe/GaMnAs bilayer (left) and Device A patterned on the GaMnAs reference layer (right). The [110] crystallographic direction and positive current direction are shown by black arrows, and angle *φ*_*H*_ indicates the directions of applied magnetic field. The bilayer structure is shown in the inset on upper left.

In order to know the properties of the GaMnAs layer itself in our composite structure, the sample was divided into two 5 × 5 mm^2^ regions, and one of the regions was then selectively etched to remove the top Fe layer. This provided the opportunity to simultaneously investigate the behavior of the Fe/GaMnAs bilayer and of the single GaMnAs layer at identical experimental conditions, so as to independently identify the magnetization reversal process in GaMnAs and in Fe layers. For transport measurements, a 1000 × 50 μm^2^ Hall device was patterned on each of these two regions by photolithography and dry etching, with the long dimension oriented along the [110] crystallographic direction of the specimen. The details of the device are shown in Fig. 1. In what follows, the Hall devices patterned from the single GaMnAs layer and from the Fe/GaMnAs bilayer will be referred to as Device A and Device B, respectively.

The primary tool which we use for investigating magnetotransport properties of the Fe/GaMnAs bilayer is the planar Hall resistance (PHR), owing to the sensitive dependence of this phenomenon on the orientation of magnetization in the ferromagnetic layers comprising the bilayer of interest. PHR measurements were performed using a sample holder which allows the magnetic field to be applied at arbitrary directions in the plane of the sample. The electromagnet used for this purpose was mounted on a rotating table, so that the field could either be swept along a chosen direction, or it could be continuously rotated in the film plane at a fixed field magnitude. The directions of the applied magnetic field *φ*_*H*_ and of the magnetizations of the magnetic layers *φ*_*M*_ are measured counterclockwise from the [110] crystallographic direction of the sample plane (i.e., from the positive current direction in the Hall device, as shown in Fig. 1).

## Results and Discussion

**Figure 2:**
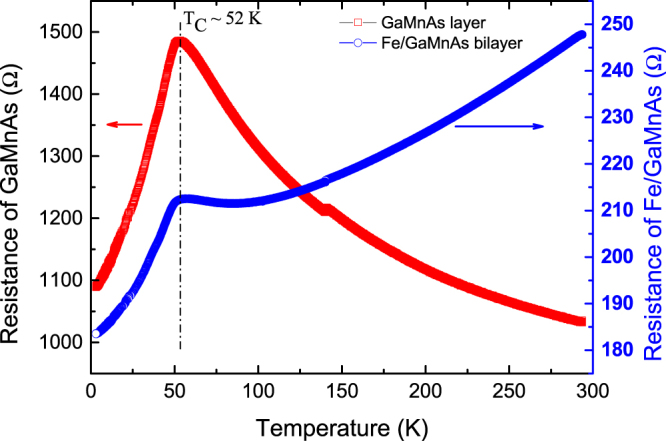
Temperature dependence of resistance measured for Device A (red squares) and Device B (blue circles). The resistance peak corresponding to transition temperature of the GaMnAs layer peaks appears near 52 K in both devices.

Temperature dependence of resistivity of GaMnAs provides a convenient means of estimating the Curie temperature of this material^19–22^, and we begin by applying this approach to both the reference sample and the bilayer. The results are plotted in Fig. 2, where red squares and blue circles show data obtained from device A and B, respectively. The temperature-dependent resistance of Device A is typical for a ferromagnetic semiconductor, with the resistance peak around 52 K providing an estimate of the Curie temperatures for the GaMnAs layer^19–22^. In case of Device B the resistance clearly shows a metallic behavior, decreasing monotonically with decreasing temperature, which indicates that the current flows predominantly through the Fe layer. However, since the resistance shows a distinct cusp near 52 K, at the same temperature as the peak observed on Device A, this indicates that electrical measurements on the Fe/GaMnAs bilayer can be used for simultaneously investigating magnetic properties of both constituent layers despite the much higher resistivity of GaMnAs.

**Figure 3:**
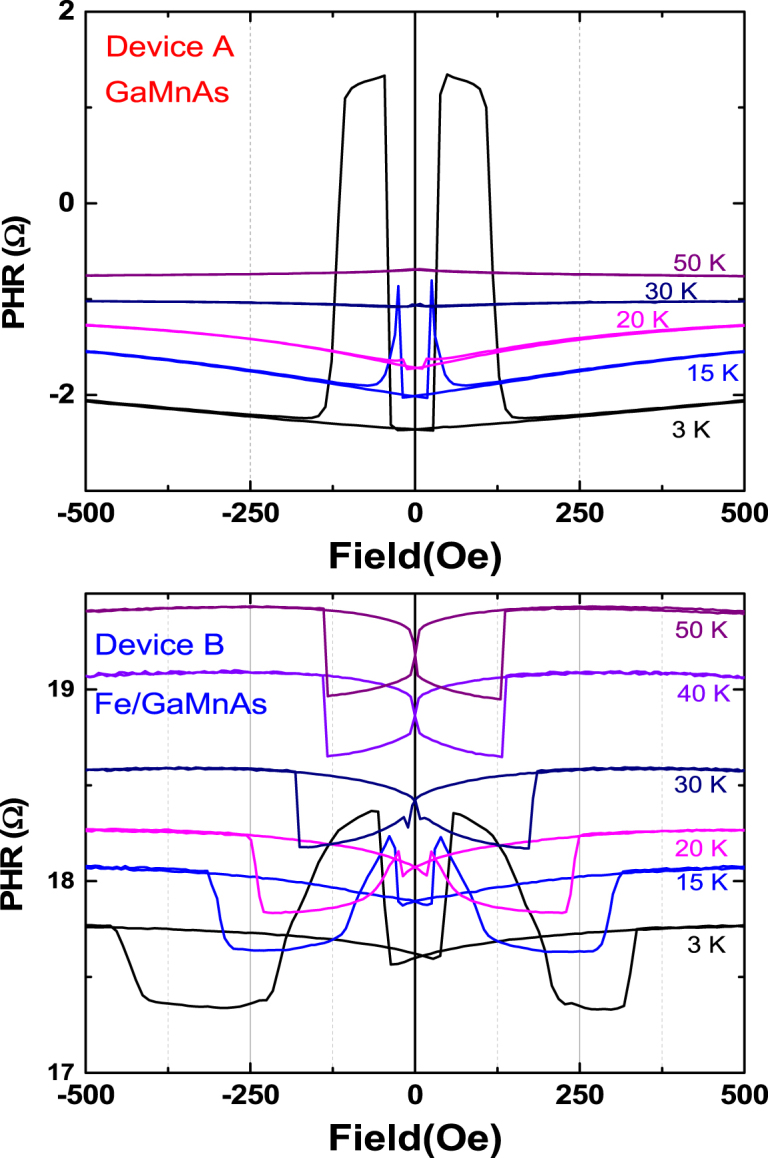
Field scan of PHR observed at *φ*_*H*_ = 80° at various temperatures. The upper and lower panels show data obtained from Device A and Device B, respectively. Device A shows typical behavior of a single GaMnAs layer, while Device B shows a complex sequence of transitions, as discussed in the text.

We then performed planar Hall resistance (PHR) measurements observed by sweeping the magnetic field along fixed field directions in the film plane. PHR data obtained in a field scans between +500 Oe and −500 Oe at *φ*_*H*_ = 80° at various temperatures are shown Fig. 3, where the top and bottom panels show data for Device A and B, respectively. The data observed on Device A (i.e., the control GaMnAs layer) show a clear two-step transition behavior at 3 K, a typical PHR behavior of a GaMnAs film with two in-plane easy axes of magnetization near the 〈100〉 directions caused by strong cubic anisotropy. However, cubic anisotropy of GaMnAs decreases very rapidly with increasing temperature, while the uniaxial anisotropy remains essentially unchanged as the temperature rises. This significantly reduces the energy barriers at the $[\bar{1}10]$ and $[1\bar{1}0]$ orientations, leading to a rotation of magnetic easy axes toward the uniaxial anisotropy direction (i.e., the $[\bar{1}00]$ and [010] easy axes move toward the $[\bar{1}10]$ direction; and the $[0\bar{1}0]$ and [100] easy axes move toward $[1\bar{1}0]$). Consequently, as the temperature rises to 15 K, the two magnetic easy directions move close to each other, so that the first and second transitions between magnetic easy directions occur very close to one another during the field scan, resulting in the appearance of the sharp peak in PHR observed at 15 K. As the temperature increase further to above 30 K, the increasing dominance of uniaxial anisotropy causes the peak to disappear altogether, since the transition now occurs directly between the uniaxial easy axes (i.e., between the $[\bar{1}10]$ and the $[1\bar{1}0]$ directions), both of which give the same PHR values. In the case of Device B, transition behavior of PHR is more complicated at low temperatures (see data for 3 K). In addition to transitions similar to those for Device A in the low field region (i.e., smaller than 200 Oe), additional transitions originating from the Fe layer appear at higher fields, as discussed below.

These PHR behavior of a ferromagnetic film is described by the equation^23,24^

1 $${R}_{PHE}=\frac{k}{t}{M}^{2}\,\sin \,2{\phi }_{M}$$

where *t* is the film thickness; *M* is its magnetization; *φ*_*M*_ is the direction of the magnetization (as indicated in Fig. 1); and *k* is a constant related to the anisotropic magnetoresistance (AMR) defined by *k* ~ (*ρ*_||_ − *ρ*_⊥_), where *ρ*_||_ and *ρ*_⊥_ are the resistivities measured when the magnetization direction is parallel and perpendicular to the current flow, respectively^25^.

The data observed on Device B at 3 K plotted in the bottom panel of Fig. 3 show transitions in opposite directions in the low and in the high field regions, indicating that the sign of *k* is opposite in the GaMnAs and in the Fe layers. This is consistent with earlier reports that the sign of *k* for GaMnAs is normally negative^25–33^, while that of Fe is positive^33–38^. The changes in PHR due to low-field transitions gradually diminish in Device B with increasing temperature, gradually becoming overcome by the Fe contribution as the Curie temperature of the GaMnAs layer is approached. This indicates that the low field transitions observed on Device B originate from the GaMnAs layer, and those in high field region (which continue to be prominent at higher temperatures) can be ascribed to the Fe layer. The fields at which these Fe transitions occur decrease rapidly with increasing temperature up to ~40 K, above which the rate of decrease slows down, as seen in the bottom panel of Fig. 3.

An interesting feature of the results obtained on Device B is the asymmetry of the coercive field of the Fe layer, appearing prominently at 3 K. We ascribe this asymmetry to exchange bias arising due to the presence of an antiferromagnetic FeO layer that forms spontaneously on the surface of the Fe layer. Even though the Fe layer was capped by a 2-nm layer of Au, it has been observed in earlier studies that oxygen can penetrate such thin Au layers to form FeO, thus resulting in exchange bias, which affects the magnetic response of the Fe layer^39,40^. Since such FeO is formed by penetration of oxygen through the thin non-uniform Au capping layer, it is disordered in composition and/or morphology^18^. This highly non-uniform and thin FeO layer is known to show a much lower transition temperature than the Néel temperature of bulk crystalline FeO^39,41^. In our system this transition occurs below 15 K, and the exchange bias effect is thus observed only in the data taken at 3 K, as shown in Fig. 3.

**Figure 4:**
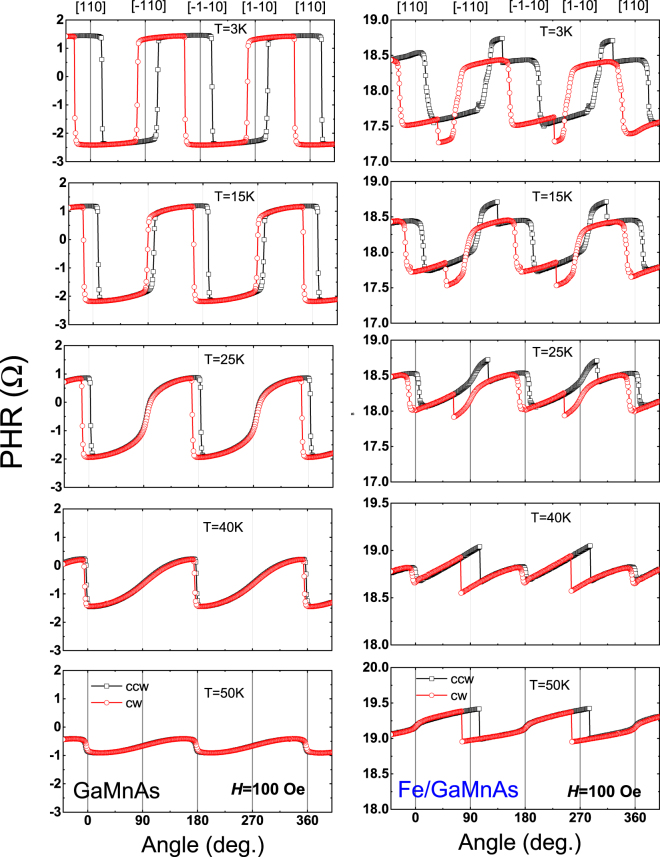
Angular dependence of PHR measured with a fixed applied field of 100 Oe at several temperatures. Results for Devices A and B are shown in left and right columns, respectively. Open squares and open circles show data obtained with CCW and CW rotations, respectively. Note the hystereses between CW and CCW rotations appearing across the 〈110〉 directions.

To further investigate the process of magnetization reversal in the bilayer, we also performed angle-dependent PHR measurements by rotating a field of a fixed magnitude at various temperatures. The PHR results measured during rotation of a 100-Oe field over 360° are shown in Fig. 4, in which the data in the left and the right columns are obtained on Devices A and B, respectively. Device A shows simple square-like hystereses between clockwise (CW) and counterclockwise (CCW) field rotations at 90° intervals, indicating hard axes at 〈110〉 crystallographic directions. The widths of the hystereses at 90° and 270° are narrower than those at 0° and 360°, indicating the presence of a uniaxial anisotropy component along $[1\bar{1}0]$ and $[\bar{1}10]$ directions, which is typical for GaMnAs films grown on GaAs substrates^42–45^. The hystereses, however, nearly disappear as temperature approaches the Curie temperature of GaMnAs layer.

The PHR behavior of Device B is much more complicated owing to the superposition of the signals from the GaMnAs and the Fe layers. One can, however, clearly see the hystereses across the 〈110〉 directions that gradually diminish with increasing temperature in a similar pattern as in Device A, that can therefore be ascribed to the GaMnAs layer. Since the GaMnAs hysteresis begin to vanish at temperatures close to *T*_*c*_, the hysteresis observed at 50 K is expected to arise from the Fe layer. Note that at 50 K (bottom panels in Fig. 4) the change of PHR observed for Device B at *φ*_*H*_ = 0° and 180° (i.e., [110] and $[\bar{1}\bar{1}0]\,\,$directions) is opposite to that for Device A. This is due to the opposite sign of *k* in the GaMnAs and Fe layers, indicating that PHR observed at 50 K in Device B is dominated by the Fe layer. Note further that the widths of the hysteresis observed for Device B at 50 K are much greater at *φ*_*H*_ = 90° and 270° than at *φ*_*H*_ = 0° and 180°, indicating the presence of strong uniaxial anisotropy along the $[\bar{1}\bar{1}0]$ and the [110] directions in the Fe layer. This orientation of uniaxial anisotropy in the Fe layer is orthogonal to the anisotropy orientation in the GaMnAs layer, as seen in the data in the left column of Fig. 4.

**Figure 5:**
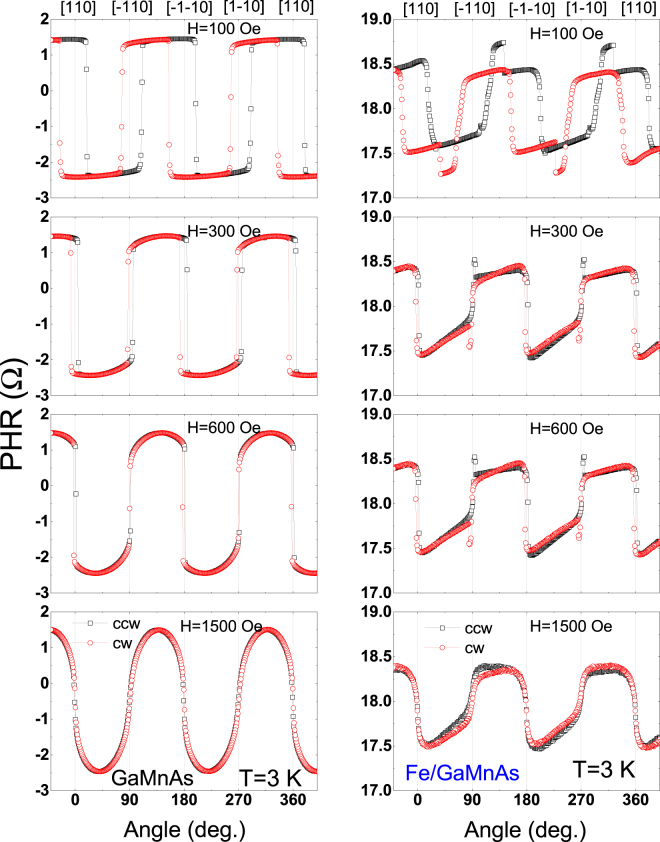
Angular dependence of PHR measured at 3 K using several different field strengths. The data from Device A and B are plotted in left and right columns, respectively. Open squares and open circles show results obtained with CCW and CW rotations, respectively. The hystereses between CCW and CW rotations diminish rapidly as the field strength increases.

To obtain additional details regarding magnetic anisotropy of the Fe/GaMnAs bilayer, we performed angle-dependent PHR measurements using different field strengths, as shown in Fig. 5, where the left and right columns again represent results for Devices A and B, respectively. In the case of Device A, the PHR hysteresis systematically decreases as the field strength increases due to increasing Zeeman effect, the angular dependence of PHR gradually becoming sinusoidal at fields over 1500 Oe. This indicates that magnetization of the GaMnAs layer experiences nearly coherent rotation above that field strength. However, in the case of Device B, the angular dependence of PHR is clearly not sinusoidal throughout the entire field range studied, indicative of the effects of the Fe layer.

To quantify magnetic anisotropy, the angle-dependent PHR of magnetic layers can be analyzed on the basis of magnetic free energy, which is described by^25,46–48^

2 $$\frac{E}{M}=\frac{1}{8}{H}_{C}\,{\cos }^{2}2{\phi }_{M}+\frac{1}{2}{H}_{U1}\,{\cos }^{2}{\phi }_{M}+\frac{1}{2}{H}_{U2}\,{\sin }^{2}({\phi }_{M}-\frac{3\pi }{4})-H\,\cos \,({\phi }_{M}-{\phi }_{H}),$$

where *H*_*C*_ is the cubic anisotropy field, *H*_*U*1_ is the well known uniaxial anisotropy field associated with the $[\bar{1}10]$direction, *H*_*U*2_ is the uniaxial anisotropy field associated with the [100] direction, *φ*_*H*_ denotes the direction of the applied magnetic field, and *φ*_*M*_ is the direction of magnetization in the film. The presence of *H*_*C*_, *H*_*U*1_, and *H*_*U*2_ in a magnetic film can be identified from angular scans of PHR, as follows. *H*_*C*_ causes the hysteresis occurring at 〈110〉 directions, accommodating the stable PHR states around the 〈100〉 directions; *H*_*U*1_ leads to differences between hysteresis widths across $[1\bar{1}0]$ and $[\bar{1}\bar{1}0]$ (as well as between $[\bar{1}10]$)^43^ and [110]); and the effect of *H*_*U*2_ leads to an asymmetric shift of the hysteresis away from the 〈110〉 directions^47,48^.

**Figure 6:**
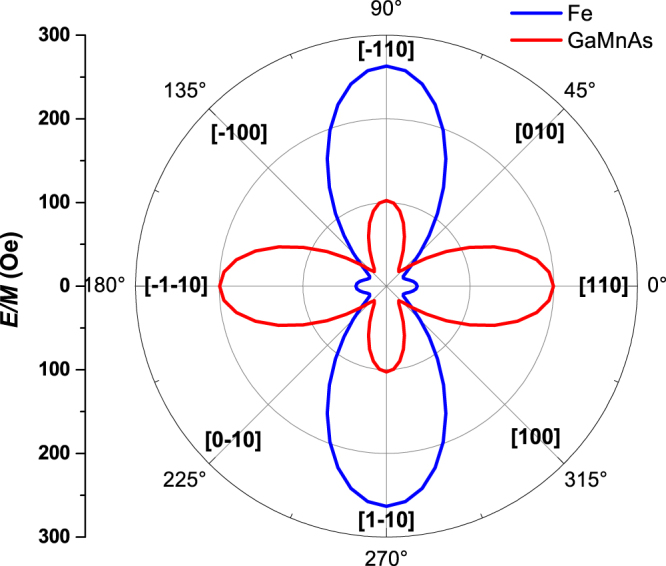
Magnetic free energy diagram for GaMnAs and Fe layers. Both layers show energy barriers at 〈110〉 directions, but with very different heights, indicating different superpositions of cubic and uniaxial magnetic anisotropy.

We use Eq. (2) to analyze the angular dependence of our PHR data, aimed at obtaining magnetic anisotropy parameters of the bilayer. The procedure for analyzing angle-dependent PHR data in terms of magnetic anisotropy are described in detail in previous publications^46–50^, and we adopted the same analysis procedures in the present case. The magnetic anisotropy parameters for the GaMnAs and the Fe layers by this approach are listed in Table 1. Using the anisotropy parameters obtained for the 3 K results, we construct magnetic free energy diagrams plotted in Fig. 6, where the red and blue lines represent the energy profiles for the GaMnAs and the Fe layers, respectively. It is now clear that both layers have four energy minima (i.e., four magnetic easy directions), which are slightly shifted away from the 〈100〉 directions due to the influence of uniaxial anisotropy *H*_*U*1_. Note further that the symmetry of uniaxial anisotropy *H*_*U*1_ is orthogonal in the two layers (i.e., it is symmetric around the $[\bar{1}10]$) direction for the GaMnAs layer, and around [110] for the Fe layer).

**Table 1:** Anisotropy fields of reference GaMnAs layer (Device A) and Fe/GaMnAs bilayer (Device B).

Device	*H*_*C*_	*H*_*U*1_	*H*_*U*2_	T (K)
A	988.3 ± 5.0	194.2 ± 2.2	18.97 ± 2.6	3
B	1566.3 ± 59.3	1073.4 ± 94.8	—	3

**Figure 7:**
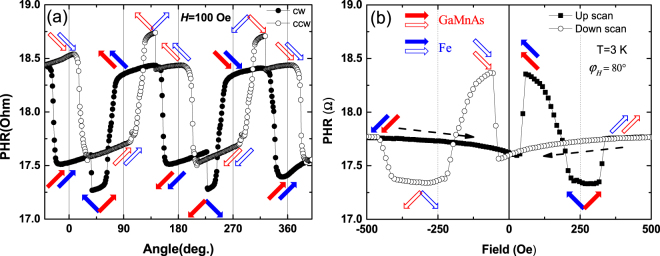
(**a**) Angular and (**b**) field scans of PHR obtained on Device B at 3 K. Open and solid symbols denote data obtained with CCW and CW rotations in (**a**) and results for up- and down- field scans in (**b**). Dotted arrows in (**b**) show directions of the field scans. Alignments of magnetization in Fe and GaMnAs layers are indicated by blue and red arrows, respectively. Parallel and perpendicular configurations between the two layers are repeatedly realized during magnetization reversal, indicating a sequence of stable PHR states.

Having established magnetic anisotropy parameters for the Fe and GaMnAs layers, we can now address the important question of magnetic alignments occurring in the two layers during magnetization reversal. To illustrate this, in Fig. 7 we plot representative angular and field scans of PHR obtained during magnetization reversal at 3 K. For the angular scan, the magnetization of the two layers was initialized by applying a field of 2000 Oe along the [100] direction (i.e., *φ*_*H*_ ≈ −45°). The field was then reduced to 100 Oe, rotated CCW by 450° (i.e., first by 360°, and by an additional 90° in order to ensure complete magnetization reversal), and returned back to −45° by CW rotation. When the field direction rotates CCW across *φ*_*H*_ = 0°, the magnetization of both layers rotates (almost simultaneously, see open symbols in Fig. 7a) by 90°, from the 4^th^ quadrant to the 1^st^ quadrant. As the field continues to rotates past 90°, transition of magnetization occurs first in the GaMnAs layer, resulting in orthogonal magnetization alignments between the GaMnAs and then Fe layers, until the Fe magnetization overcomes its relatively large energy barrier (see Fig. 6) and restores parallel alignments between the two layers. This pattern of parallel and orthogonal alignments between the GaMnAs and Fe layers, indicated by open red and blue arrows in Fig. 7(a), is repeated as the magnetization reversal continues. A similar progression of parallel and orthogonal alignments (indicated by solid arrows in the figure) is observed for CW rotation of the field. Importantly, in regions between transitions (i.e., when magnetizations in both layers remain either parallel or orthogonal), represent stable PHR states, and are thus of interest for logic operations.

For the field scan measurements shown in Fig. 7(b), the field direction was set to 80° and scanned between +500 Oe and −500 Oe. A down-scan from positive to negative fields at this orientation leads first to a nearly simultaneous CW rotation of magnetization in both layers, retaining parallel alignment, followed by a subsequent an orthogonal alignment after the GaMnAs magnetization makes another 90° transition while the Fe layer remains unchanged, as shown by red and blue arrows in Fig. 7(b). A similar progression, again in the form of CW magnetization transitions, occurs during the upward scan of the field, shown by full symbols in Fig. 7(b). As can be seen from the field-scan behavior, magnetization reversal driven by the field also elicits a similar series of stable PHR states as observed during field rotation.

**Figure 8:**
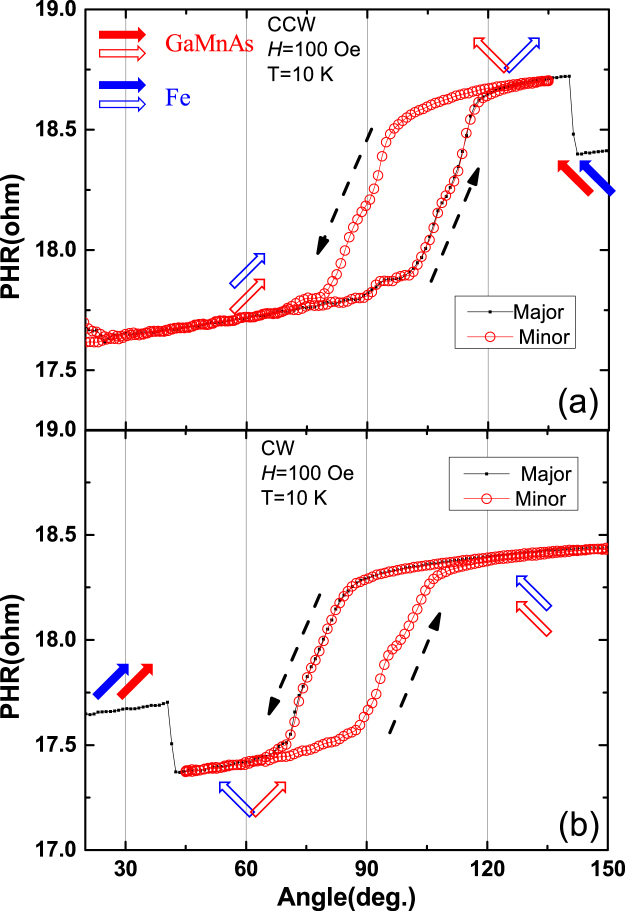
Minor PHR hysteresis loop scans between CCW and CW rotations measured for Device B at 10 K. Major loop data obtained by CCW and CW rotations are plotted using small black symbols in upper and lower panels, respectively, and corresponding minor loop results are shown as open red circles. Dotted arrows indicate angular scan directions of the minor loops. Magnetization alignments occurring in Fe and GaMnAs layers during the angular scans are shown by blue and red arrows, respectively. Note that the minor hysteresis loops in both cases shift toward the orthogonal configuration of magnetizations in GaMnAs and Fe layers, indicating ferromagnetic IEC between the two magnetic layers.

Having identified magnetic configurations for sequential stable PHR states in the bilayer, we finally inquire whether or not the GaMnAs and Fe layers interact through interlayer exchange coupling (IEC). In order to observe the effects of IEC, we performed minor-loop hysteresis scans in the magnetic field region in which only the magnetization of the GaMnAs layer experiences a 90° rotation, while the magnetization of Fe layer remains unchanged. Such minor loops measured at 10 K are plotted in Fig. 8, in which the data in the upper and lower panels are obtained after completing major hysteresis loops by CCW and CW rotations of the field, respectively. One can clearly see that the minor hysteresis loops are shifted in opposite directions around the *φ*_*H*_ = 90° field orientation for the two cases (i.e., they shift to larger angles in the upper panel, and to lower angles in the lower panel). Note, however, that the shift of the minor hysteresis loop in both cases occurs toward the perpendicular configuration between magnetizations in the two magnetic layers (as marked by empty red and blue arrows). This indicates that the 90° rotation of magnetization in the GaMnAs layer is harder to achieve when it occurs from parallel to perpendicular configuration, and is easier to return from perpendicular to parallel alignments. Such preference for parallel magnetization alignment in the bilayer indicates the presence of ferromagnetic (FM) IEC between the two magnetic layers. This observation of FM IEC between GaMnAs and Fe layers is consistent with results obtained from the same type of Fe/GaMnAs bilayer by other experimental techniques such as SQUID and XMCD measurements^15,16,18^.

Even though the proximity effects occur at the interface region, they can affect the global magnetic behavior of GaMnAs layer as a whole, as follows. The magnetization of the GaMnAs/ferromagnet (FM) bilayer in the presence of interlayer exchange coupling can be described in terms of the exchange spring model, in which magnetization of the GaMnAs layer rotates continuously as a function of distance from the interface, caused by the coupling with the magnetization of the neighboring FM layer^51^. Such magnetic coupling then governs the behavior of the entire GaMnAs layer, affecting the reversal of magnetization in the GaMnAs layer as a whole. The effect of magnetic coupling can thus be observed in PHR, as it measures the magnetization reversal of entire GaMnAs layer.

## Summary and Conclusions

We have used planar Hall resistance (PHR) measurements to investigate magnetic anisotropy of a hybrid bilayer structure comprised of adjacent Fe and GaMnAs ferromagnetic layers. The PHR results revealed that the anisotropic magnetoresistance (AMR) has opposite signs in the two magnetic layers. The PHR data has also shown that the cubic and uniaxial anisotropies in both layers can be described by two nearly orthogonal in-plane easy axes that, however, are not coincident. Careful analysis of minor hysteresis loops has revealed the presence of clear ferromagnetic exchange coupling between the Fe and GaMnAs layers. Thus, although the magnetizations in the two layers undergo rotations between their respective easy axes separately, those rotations are influenced by the presence of the adjacent ferromagnetic layer. Furthermore, because of different magnetic anisotropies and coercive fields characterizing the two layers, rotations of magnetization in the layers are not simultaneous, thus resulting in a series of consecutive parallel and orthogonal configurations as the field is swept or rotated. This in turn leads to a series of distinct stationary states of PHR. Importantly, this magneto-transport investigation of diverse but stable configurations of magnetization alignment in adjacent Fe and GaMnAs layers demonstrates the potential for applications of such hybrid Fe/GaMnAs structures in multistate magnetic memory devices.
